# fNIRS-based early identification of mild cognitive impairment: a large-scale multi-paradigm study with ensemble machine learning models

**DOI:** 10.3389/fneur.2026.1738099

**Published:** 2026-03-03

**Authors:** Yufei Chong, Can Duan, Xinzi Xu, Zhengliang Li, Heling Zhang, Jingyi Gong, Qingqing Wu, Lirong Xia, Peiwen Zhang, Wenguang Xia

**Affiliations:** 1 The Affiliated Hospital of Hubei Provincial Government/Hubei Rehabilitation Hospital, Wuhan, China; 2 Hubei Engineering Research Center of Neuromodulation technology, Wuhan, China; 3 Hubei University of Chinese medicine, Wuhan, China; 4 Hubei Provincial Hospital of Integrated Traditional Chinese and Western Medicine, Wuhan, China; 5 Hubei Provincial Clinical Research Center for Stroke Rehabilitation of Integrated Traditional Chinese and Western Medicine, Wuhan, China

**Keywords:** fNIRS, Alzheimer’s disease, ADRD, 1-back task, machine learning models

## Abstract

**Background:**

Early and accurate identification of mild cognitive impairment (MCI) is crucial for timely intervention and preventing further cognitive decline. Functional near-infrared spectroscopy (fNIRS) is a non-invasive, portable tool for clinical screening, but its diagnostic accuracy is often constrained by single-paradigm approaches and small sample sizes. To address this limitation, this study aimed to develop and validate an efficient early MCI screening model by integrating large-sample fNIRS data from resting-state and 1-back task paradigms using ensemble machine learning, thereby enhancing the accuracy and reliability of early MCI diagnosis.

**Methods:**

A total of 462 right-handed participants (185 MCI patients and 277 healthy controls, aged 58 -87 years) were included in the final analysis after screening, with MCI diagnosis jointly determined by two experienced neurologists based on Petersen’s criteria. fNIRS signals were collected during resting-state and 1-back task sessions; after preprocessing in MATLAB, features were extracted from oxygenated hemoglobin (HbO) signals of both paradigms.

**Results:**

Feature selection was performed via a gradient boosting classifier based on feature importance scores, resulting in 108 selected features. Five classifiers were trained and evaluated using 10-fold cross-validation. The integrated dataset combining resting-state and 1-back task features outperformed the single-paradigm datasets: the Neural Network model on this integrated dataset achieved an accuracy of 86.49%, sensitivity of 94.74%, specificity of 77.78%, and Area Under the Curve (AUC) of 93.49%. In contrast, the Nearest Neighbor model on the resting-state dataset and the Decision Tree model on the 1-back task dataset yielded accuracies of 70.27% and 75.68%, respectively. Group classification using MoCA scores achieved an accuracy of 86.55%, which was comparable to single-paradigm machine learning models but inferior to the integrated model.

**Discussion:**

This study demonstrates the value of a large-sample, data-driven approach and multi-paradigm feature integration in fNIRS-based MCI screening, providing an efficient diagnostic model for clinical application.

**Clinical trial registration:**

https://www.chictr.org.cn/showprojEN.html?proj=192047.

## 1 Introduction

The number of people living with Alzheimer’s disease and related dementias (ADRD) is projected to increase significantly, with estimates suggesting over 150 million cases by 2050 due to population aging (1). Alzheimer’s disease (AD), the primary neurodegenerative condition contributing to ADRD, leads to cognitive decline, functional impairment, and behavioral changes, resulting in stigma, social isolation, and considerable social and economic burdens (2). As of 2019, the global economic burden of ADRD was $2.8 trillion, with projections indicating it will rise to $16.9 trillion by 2050, presenting considerable challenges due to its subtle onset and relentless progression (3). Mild cognitive impairment (MCI) represents a transitional phase between normal cognitive aging and early dementia. An estimated 15.4–33.4% of individuals with MCI progress to dementia annually, while others remain stable for prolonged periods, sometimes for more than 10 years (4, 5). Notably, about 16% of individuals diagnosed with MCI return to normal or near-normal cognition within 1 year (6). Although there are several potential treatments for AD, such as enzymes that reduce amyloid-*β* production and antibodies that help clear amyloid-β from the brain, at present, no medications are capable of completely curing dementia or making substantial changes to its clinical progression (7). Moreover, early detection of MCI is essential for timely intervention and the potential prevention of further cognitive decline, which has been shown to restore normal cognitive function in up to 40% of older adults (8). Therefore, it is essential to predict the onset of MCI in older adults as soon as possible and proactively assist in preventing or delaying the progression of MCI and AD.

MCI diagnosis generally necessitates a multidisciplinary approach, involving clinical assessments, neuropsychological evaluations, advanced imaging techniques, and biomarker analysis (9). Specifically, traditional MCI screening mainly relies on neuropsychological evaluations such as the Mini-Mental State (MMSE) and the Montreal Cognitive Assessment (MoCA), which assess cognitive abilities through question-and-answer tasks and operational tasks (10). However, the results are significantly influenced by an individual’s education level and cultural background, limiting their value as screening tools for cognitive impairment. While neuroimaging modalities like magnetic resonance imaging (MRI) and positron emission tomography (PET) offer objective biomarkers, their high cost and limited accessibility preclude routine, large-scale screening (11). Consequently, there is a pressing need for an objective, cost-effective, and scalable tool to facilitate early MCI detection in community and clinical settings.

Functional near-infrared spectroscopy (fNIRS) emerges as a promising candidate to address this need. It utilizes near-infrared light (600–900 nm), which penetrates the scalp and skull to reach the cerebral cortex. This non-invasive optical neuroimaging technique measures cortical hemodynamic changes by detecting concentration changes in oxyhemoglobin (HbO) and deoxyhemoglobin (HbR). The detection principle is based on neurovascular coupling, a key physiological mechanism in the brain. When patients engage in cognitive tasks, neurons in specific brain regions become active, consuming local HbO, which is converted into HbR. Notably, aberrant neurovascular coupling has been implicated in the pathogenesis of cognitive deficits in neurodegenerative diseases such as AD. Compared to fMRI, fNIRS offers superior temporal resolution, portability, lower cost, and greater tolerance to motion artifacts, making it particularly suitable for dynamic cognitive assessments in clinical and naturalistic settings (12).

A growing body of fNIRS research has revealed distinctive neural signatures in MCI. For instance, research on brain network dynamics has found that metrics like dynamic resting-state functional connectivity (dRSFC) demonstrate greater sensitivity than static resting-state functional connectivity (sRSFC) strength in differentiating MCI from healthy controls (HCs) (13). Furthermore, fNIRS studies comparing brain activation patterns of MCI patients and healthy controls (HCs) during the Stroop task have found that both groups show enhanced cortical activation in the dorsolateral prefrontal cortex (dPFC), ventrolateral prefrontal cortex (VLPFC), and parietal lobe (PL). Notably, the MCI group shows compensatory activation in the VLPFC and PL during the late stage of the task. This finding suggests that despite having longer reaction times, MCI patients can compensate for cognitive impairments through prefrontal and parietal activation at a partial behavioral level (14). Additionally, some studies have combined fNIRS with other techniques like electroencephalography (EEG) to gain a more comprehensive understanding of neural activity in MCI (15). However, existing fNIRS studies on MCI are characterized by two main limitations. First, they often focus either on task-evoked functional activation or on resting-state network properties, rarely integrating both to provide a complete picture of brain dysfunction. Second, and more critically, many findings are derived from single-paradigm experiments with relatively small sample sizes, which may contribute to inconsistent results and limit generalizability.

These limitations underscore the need for analytical methods capable of handling multi-paradigm data from larger cohorts. Traditional statistical methods (e.g., t-tests, ANOVA), while useful for simple comparisons, are not optimal for capturing the complex, nonlinear patterns in high-dimensional fNIRS data and often underutilize the available information.

Machine learning (ML) offers a powerful alternative for fNIRS data analysis. Models such as linear discriminant analysis (LDA), support vector machines (SVM), multi-layer perceptrons (MLP), and convolutional neural networks (CNN) are capable of handling large, high-dimensional datasets. Techniques including recursive feature elimination (RFE) and information-gain-based feature selection help identify key features and optimize model parameters, thereby improving the accuracy of MCI recognition (16). Furthermore, multi-model fusion strategies, such as Bagging and Boosting, integrate the strengths of different algorithms. For instance, while linear models excel in processing linear relationships, deep learning models are more adept at identifying complex, non-linear patterns. By leveraging these integrated approaches, it is possible to capture abnormal brain activity in MCI patients more accurately, which enhances early detection and facilitates the translation of MCI research from theory to clinical application.

Therefore, this study integrates resting-state and task-state fNIRS data from a large cohort of 525 elderly participants. By employing multi-paradigm feature extraction and extensive machine learning modeling, we aim to develop and validate an optimal model for the early identification of MCI, thereby contributing a more robust framework for early screening.

## 2 Method

### 2.1 Participant

**Figure 1:**
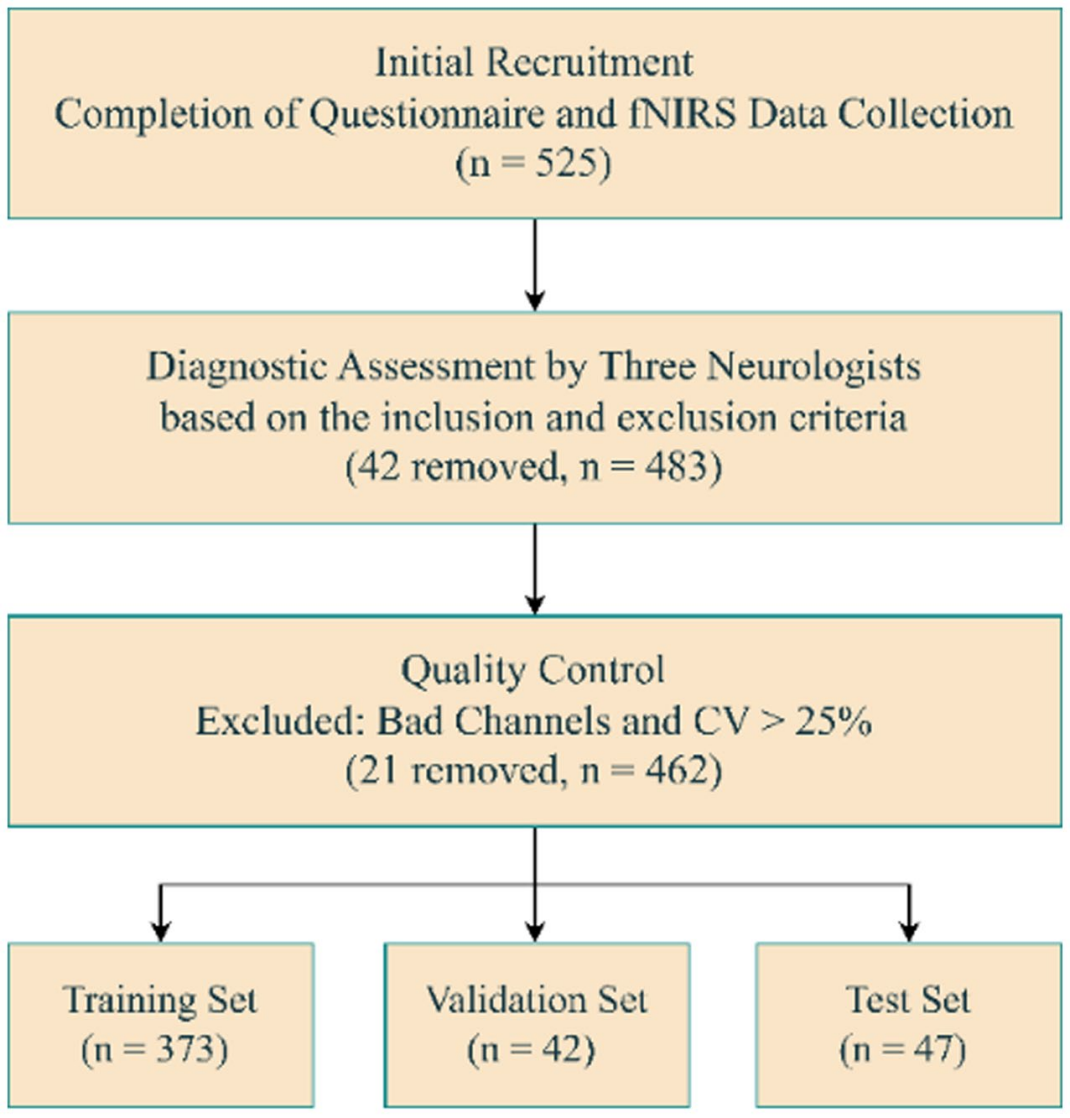
Data screening and dataset splitting.

A total of 525 right-handed participants, aged between 58 and 87 years, were recruited for this study (Figure 1). The MCI patients were recruited from Hubei Province Directly Affiliated Institution Hospital (Hubei Rehabilitation Hospital) from August 2023 to July 2024. The HCs were recruited from the local community through advertisements. Before the start of the experiment, written informed consent was obtained from each participant. This study was approved by the Ethics Committee of Hubei Rehabilitation Hospital.

All subjects underwent a standardized clinical evaluation, including a medical history interview and a series of neuropsychological tests. These tests included the Chinese version of the mini-mental state examination (MMSE), the Beijing version of Montreal cognitive assessment (MoCA), the Shape Trail Test (STT), the Digit Span Test (DST), the Instrumental Activities of Daily Living Scale (IADL), the Pittsburgh Sleep Quality Index (PSQI), the Symptom Checklist 90 (SCL-90), and the social support rating scale (SSRS).

The diagnosis of MCI was established through a standardized, consensus-based procedure in which the clinical judgment of neurologists served as the definitive criterion. First, two experienced neurologists made a preliminary diagnosis based on the core clinical criteria proposed by Petersen et al. (2001, 2004). These criteria required the concurrent presence of: (1) a memory or cognitive complaint confirmed by an informant;(2) normal activities of daily living or slight impairment in instrumental activities of daily living; (3) cognitive decline in a single domain or multiple domains; and (4) no dementia symptoms.

To objectively assess and quantify cognitive decline (criterion 3), MoCA and other standard neuropsychological tests were administered. Specifically, the MoCA score cutoff points determined by Huashan Hospital were as follows: 19 for individuals with no more than 6 years of education, 22 for those with 7 to 12 years of education, and 24 for those with more than 12 years of education. To ensure diagnostic rigor, explicit rules were followed: a MoCA score at or below the cutoff supported the clinical diagnosis. If a MoCA score fell within two points above the cutoff, or if the MoCA result was inconsistent with the clinical impression, the case underwent an additional independent review by the same neurologists. Following this review, a final diagnosis was established in accordance with the Diagnostic and Statistical Manual of Mental Disorders, Fifth Edition (DSM-5) criteria for mild neurocognitive disorder. This final diagnosis served as the definitive ground-truth for group assignment.

The inclusion criteria for HCs were: (1) no complaint of memory or other cognitive impairment, (2) normal performance on neuropsychological tests, adjusted for age, gender and education, (3) no significant impairment in activities of daily living, and (4) no severe visual or auditory impairment. All the participants were excluded if they had: (1) a clear history of stroke; (2) a history of psychosis or congenital mental retardation; (3) a history of substance addiction, e.g., drugs, alcohol; (4) the presence of psychological symptoms or severe sleep disorders (SCL-90 score ≥ 160 points or PSQI score ≥ 16 points); (5) other nervous system diseases that could cause cognitive impairment.

### 2.2 Measures

#### 2.2.1 Experimental design

During the resting-state data collection, participants were seated comfortably in a chair. The session consisted of two periods: a 30-s baseline period, during which participants fixated on a “+” displayed on the screen, and a 5-min resting period, where participants were asked to close their eyes and relax.

**Figure 2:**
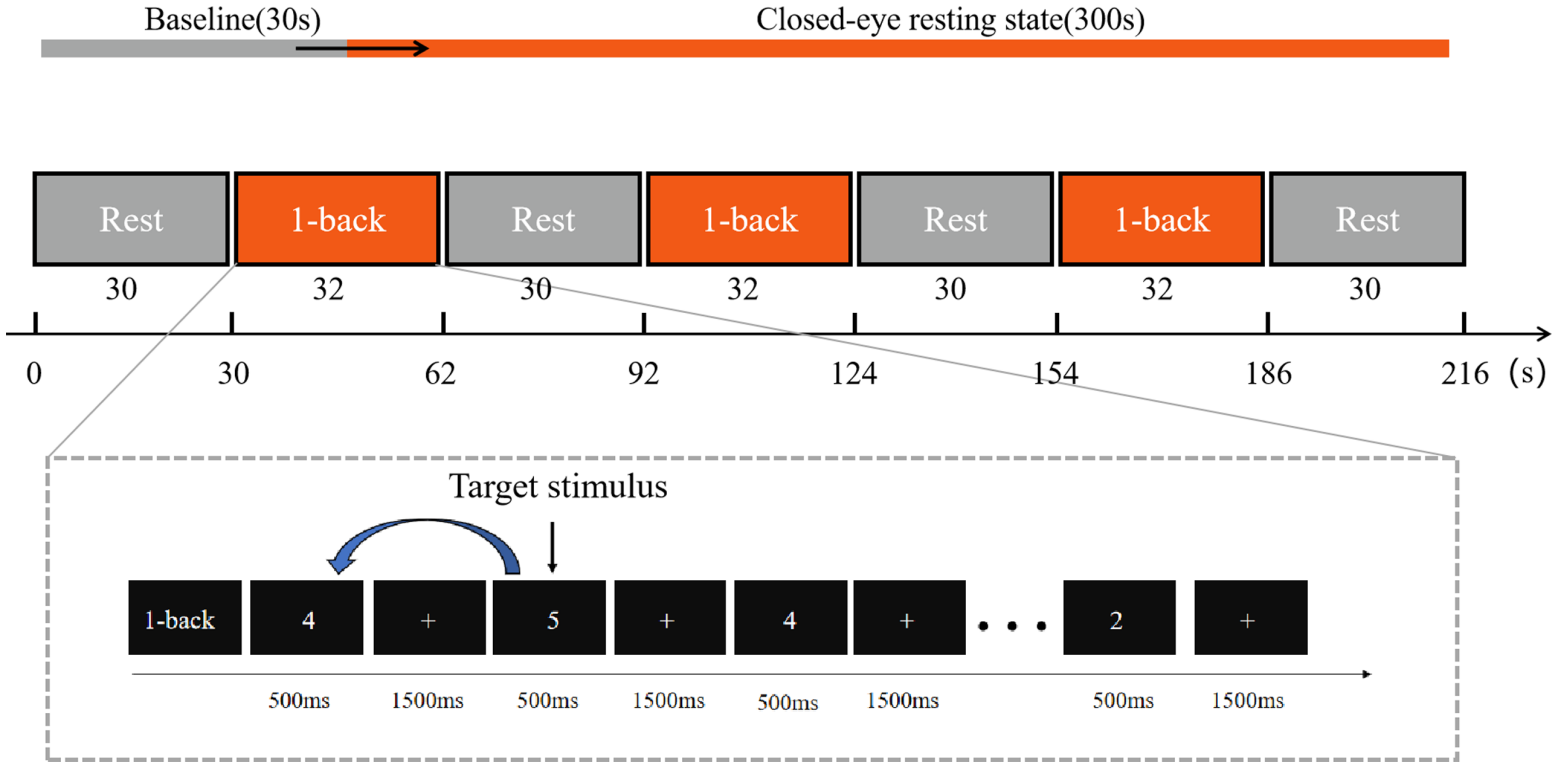
Resting-state data collection process.

The entire task consisted of four 30-s resting baseline periods and three 32-s 1-back task periods, as illustrated in the Figure 2. During the baseline periods, participants were instructed to fixate on a central “+” symbol displayed on the screen. In the task periods, participants were required to observe a series of numbers presented on the screen and make a judgment for each number regarding whether it matched the previously presented number. Each task block comprised 15 trials, with 4 target trials (target probability = 27%). Each number was displayed for 500 ms, followed by a 1,500 ms interstimulus interval during which the “+” symbol reappeared on the screen. The detailed task flow is shown in the figure below.

#### 2.2.2 fNIRS measurement

**Figure 3:**
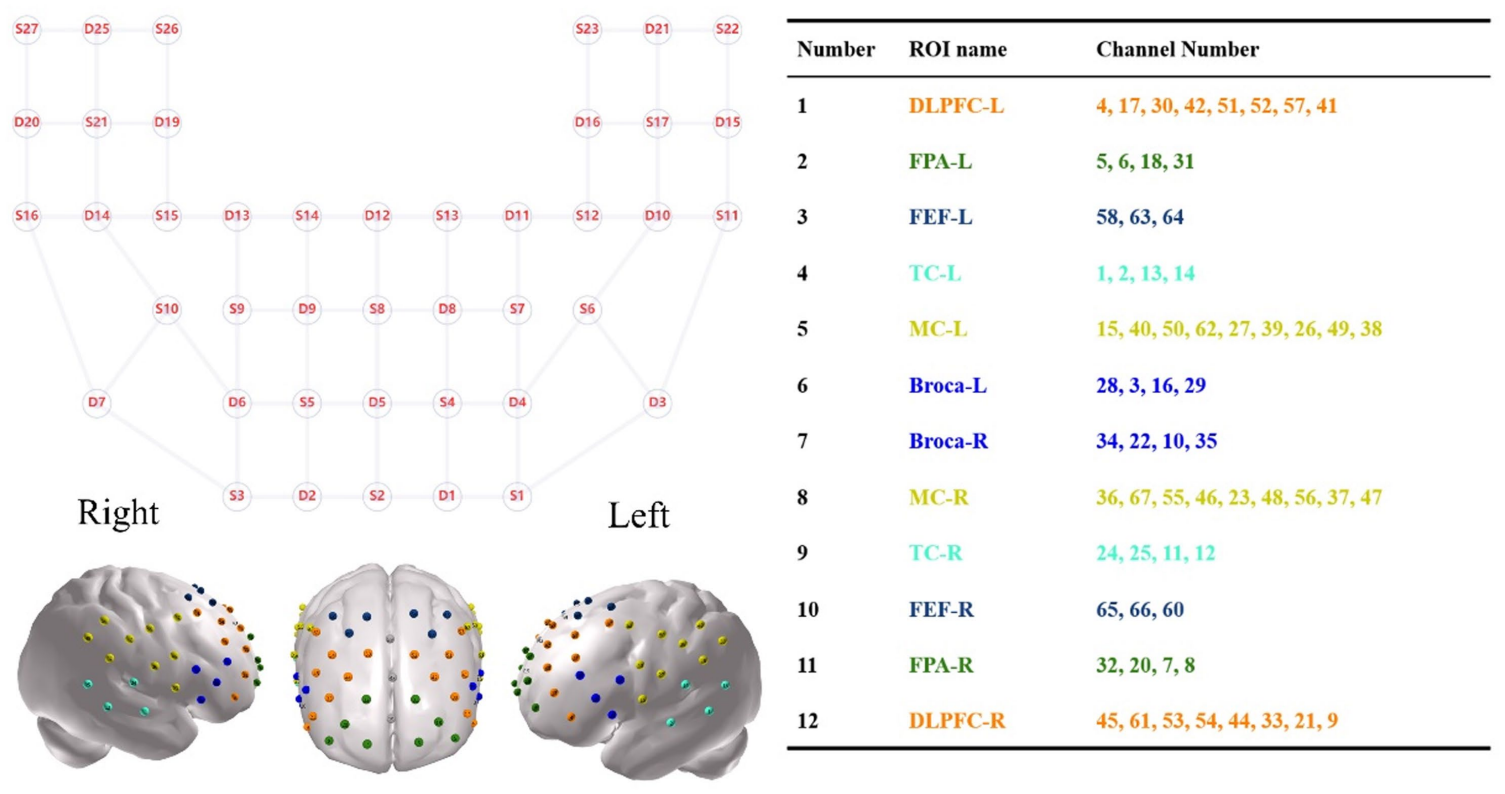
Spatial distribution of 67 fNIRS channels in 2D and 3D views.

A multichannel functional near-infrared spectroscopy (fNIRS) system (BS-7000, Wuhan Znion Technology Co., Ltd., Wuhan, China) was used to collect fNIRS signals at 690 nm and 830 nm with a sampling rate of 20 Hz. The system included 27 sources and 25 detectors, forming 67 channels with a 3 cm source-detector separation. Optodes were positioned following the international 10–20 system, with S2 aligned to Fpz. Spatial coordinates of key anatomical landmarks (Nz, Cz, AL, RL) and optode positions were recorded using a 3D digitizer. Channel locations were mapped onto the Montreal Neurological Institute (MNI) space using NIRS-SPM25 (17, 18), and categorized into twelve regions of interest (ROIs) based on Brodmann areas (see Figure 3).

Channels were categorized into 12 ROIs based on the maximum overlap probability, including six symmetrical regions: dorsolateral prefrontal cortex (DLPFC), frontal pole area (FPA), frontal eye field (FEF), temporal cortex (TC), motor cortex (MC), and Broca’s area.

### 2.3 Data analysis

#### 2.3.1 fNIRS data analysis

fNIRS data analysis was performed in MATLAB 2014a. Quality control identified bad channels with a coefficient of variation (CV) > 25%, and datasets with >25% bad channels were excluded (19), resulting in the removal of 21 participants. Batch preprocessing used the Homer2 toolbox (20), including: (1) converting raw optical intensity to optical density (OD); (2) detecting motion artifacts (tMotion = 0.5 s, STDEVthresh = 20, tMask = 3 s, AMPthresh = 5) (21) and correcting them by spline interpolation (*p* = 0.99); (3) band-pass filtering (0.01–0.1 Hz) to reduce physiological noise; and (4) converting OD to relative concentrations accordance with the modified Beer–Lambert Law (22). For the 1-back task, three blocks were averaged to ensure a robust hemodynamic response, each block consisting of a 10-s pre-task and a 50-s post-task interval.

#### 2.3.2 Features extraction

For further analysis, oxygenated hemoglobin (HbO), known for its high sensitivity to cerebral blood flow (23), was processed in the NIRS_KIT toolbox (24). To comprehensively describe brain states, features were extracted from both resting and task-based paradigms. Task-related features included GLM-derived beta values and key hemodynamic metrics (mean, integration, peak amplitude, slope). Resting-state features focused on functional connectivity (FC) and graph-theoretical metrics. FC including time-domain functional connectivity (Pearson correlation) and wavelet coherence (WCO) based on wavelet-transformed signals to quantify frequency-specific (0.01–0.08 Hz) correlations, consistent with previous resting-state fNIRS studies (25–27). Graph-theoretical metrics were calculated using the GRETNA toolbox (28), including Degree, Clustering Coefficient, Global/Local Efficiency, Degree Centrality, Betweenness Centrality, Shortest Path Length, and Small-World Properties (29, 30). Multi-channel fNIRS analysis involves channel-level analysis, which provides high spatial resolution, and ROI-level analysis, which enhances robustness by averaging signals across broader regions (21, 31). To address the uncertainty in detecting MCI-related neural patterns, both feature sets were incorporated into machine learning models.

#### 2.3.3 Machine leaning analysis feature selection

**Figure 4:**
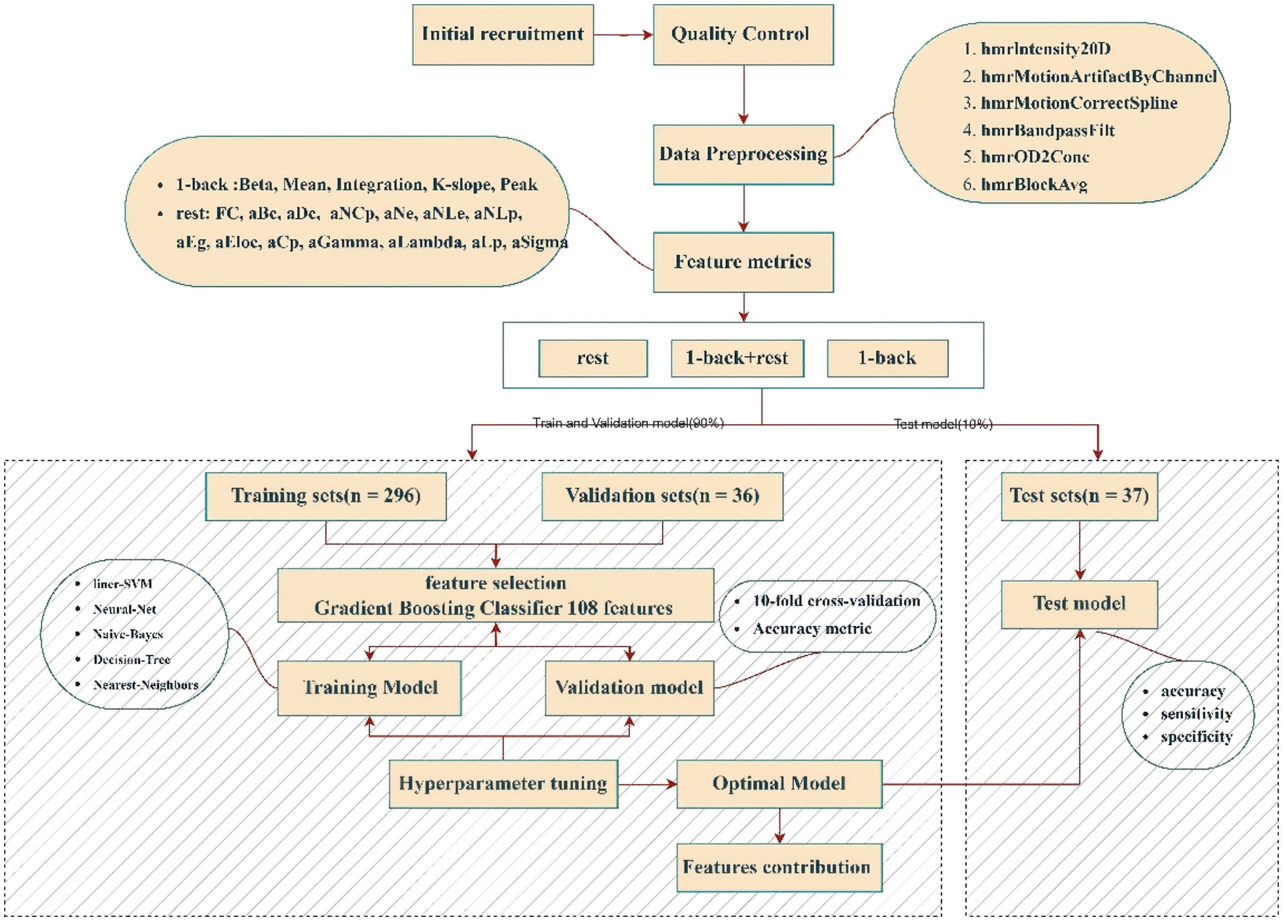
Data processing pipeline.

To achieve early screening of mild cognitive impairment (MCI), we constructed machine learning models based on fNIRS data from resting state and 1-back task paradigms. A total of 5,529 resting-state and 395 task-related features were extracted to develop models for each paradigm and a combined model integrating both datasets. Three datasets were analyzed: resting-state, 1-back, and an integrated feature set. Gradient Boosting Classifier (GBC) was employed for feature selection (32, 33), identifying the top 108 most relevant features based on importance scores. Specifically, a GBC model with 20 estimators was trained solely on the training subset to compute feature importances, and the features were then ranked and selected according to these scores. GBC, an ensemble learning method, builds successive decision trees, with each tree correcting the errors of the previous one, creating a highly predictive model (34). This approach excels in capturing complex feature interactions and robustly ranks feature importance (Figure 4).

#### 2.3.4 Model training and evaluation

All models were implemented in Python using scikit-learn, leveraging its comprehensive suite of classifiers and evaluation tools widely applied in machine learning. Given the imbalance between the MCI and HC groups, random over-sampling was applied to the minority MCI class in the training set to achieve a balanced class distribution (1:1) (35). The resampling procedure was implemented using the resample function in scikit-learn and was restricted to the training data only to prevent information leakage. For the selected 108 features, five commonly classifiers (Linear Support Vector Machine, Neural Network, Naive Bayes, Decision Tree, and k-Nearest Neighbor) were employed. The underlying principles and performance of these classifiers have been extensively discussed in the literature (36). Hyperparameter optimization was performed using GridSearchCV, which systematically explored candidate hyperparameter values through 10-fold cross-validation conducted exclusively on the training set (37). This inner cross-validation ensured robust selection of the optimal configuration while preventing overfitting to the validation data.

Accuracy served as the primary evaluation metric, with 10-fold cross-validation ensuring robust performance estimation and mitigating overfitting. The best-performing model, identified through this process, was retrained on the full test set for final evaluation. The optimized model was evaluated on an independent test set to assess generalization performance. Accuracy, sensitivity and specificity were used as performance metrics to evaluate the model’s ability to correctly detect MCI, differentiate healthy controls, and maintain overall predictive reliability. This approach ensured an unbiased performance assessment while confirming the model’s robustness across unseen data (38).

#### 2.3.5 Statistics analysis

This study first assessed the differences between clinical characteristics and fNIRS features across MCI and HC groups within the training set using independent sample *t*-tests, and calculated the correlation between MoCA scores and each fNIRS feature through Pearson correlation analysis. The *p*-values from the *t*-tests were used to evaluate the significance of group differences, while the Pearson correlation coefficients (*r* values) reflected the strength of the association between each feature and cognitive function. After constructing the optimal model, feature contribution analysis during model training was used to calculate the contribution weights of the selected features. By comparing the results of the *t*-test, correlation analysis, and feature contribution analysis, the predictive value of the features chosen in the model was further validated, along with their alignment with clinical differences and their correlation with cognitive function, ensuring the effectiveness of the selected features in the classification task.

## 3 Result

### 3.1 Demographic and clinical characteristics

A total of 462 participants were included in this study. The training and cs were derived from the same recruitment sample at a 9:1 ratio, resulting in 373 subjects (149 MCIs, 224 HCs) for training and 42 subjects (17 MCIs, 25 HCs) for validation. An independently recruited test set, representing 10% of the total sample, included 47 subjects (19 MCIs, 28 HCs), which was fully held out from feature selection, and model training. The demographic characteristics of participants in each group are shown in Table 1. Except for MoCA scores (*t* = 25.046, *p* < 0.001), no significant between-group differences in clinical characteristics, including sex (*χ*^2^ = 0.554, *p* = 0.475), age (*t* = 1.070, *p* = 0.285), and education (*t* = −1.390, *p* = 0.165).

**Table 1:** Demographic characteristics.

Subject characteristics	MCI		HC		Test statistics
	*N*	Mean (SD)	*N*	Mean (SD)	
Sex (male/female)	185	56/129	277	93/184	*χ*^2^ = 0.554, *p* = 0.457
Age (years)	185	72.45 ± 5.92	277	71.90 ± 4.69	*t* = 1.070, *p* = 0.285
Education	185	11.44 ± 3.91	277	11.92 ± 3.63	*t* = −1.390, *p* = 0.165
MoCA	185	21.60 ± 2.26	277	26.34 ± 1.79	*t* = −25.046, *p* < 0.001
ACC (%)	185	59.50 ± 22.17	277	71.31 ± 20.92	*t* = −5.661, *p* < 0.001
RT (ms)	185	805.02 ± 197.45	277	763.78 ± 171.03	*t* = 2.33, *p* = 0.020

### 3.2 Performance of machine learning models

**Figure 5:**
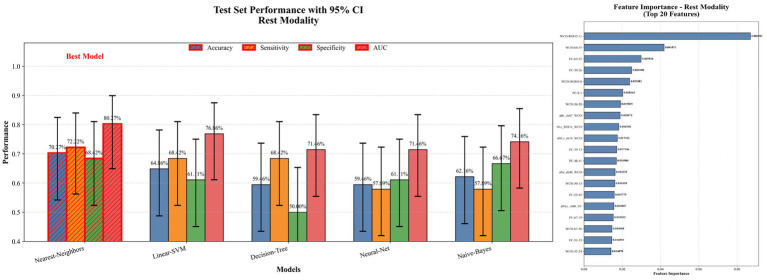
Model performance and feature importance on rest dataset.

**Figure 6:**
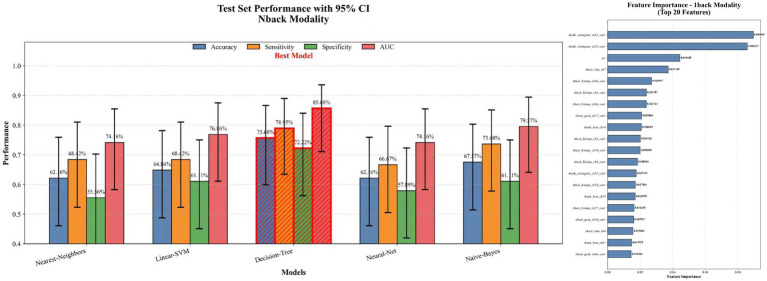
Model performance and feature importance on 1-back task dataset.

**Figure 7:**
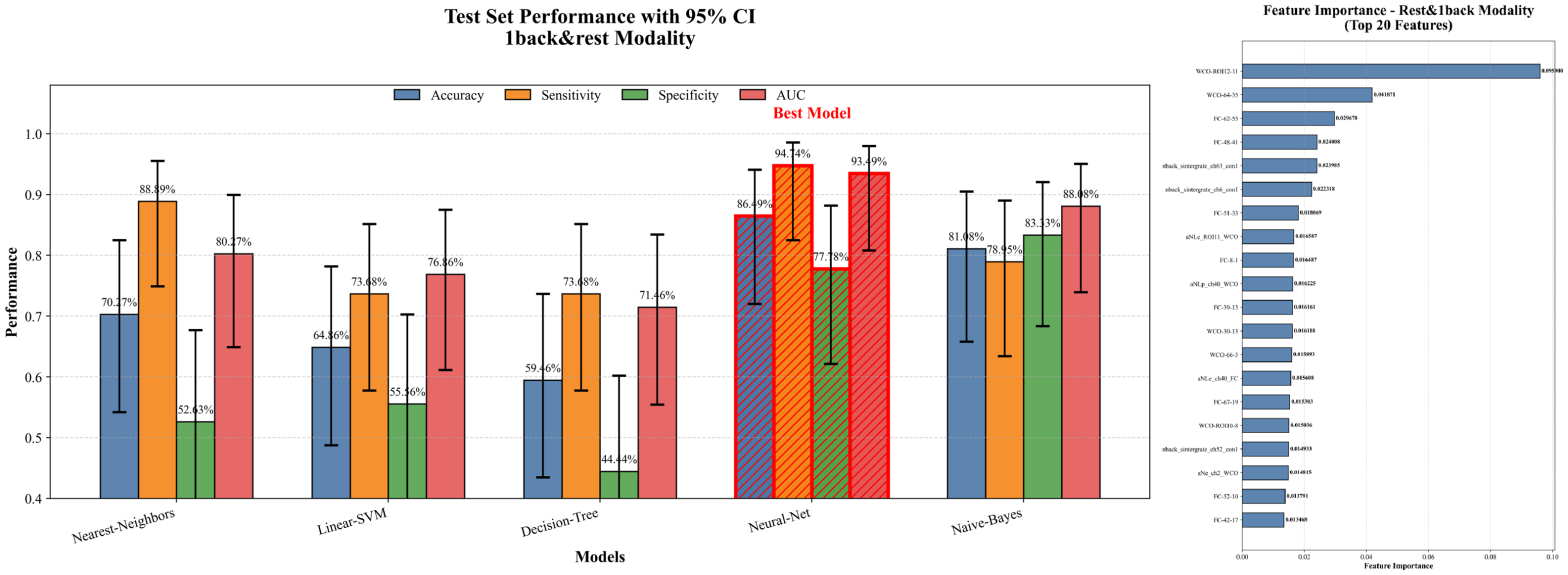
Model performance and feature importance on rest and 1-back dataset.

**Figure 8:**
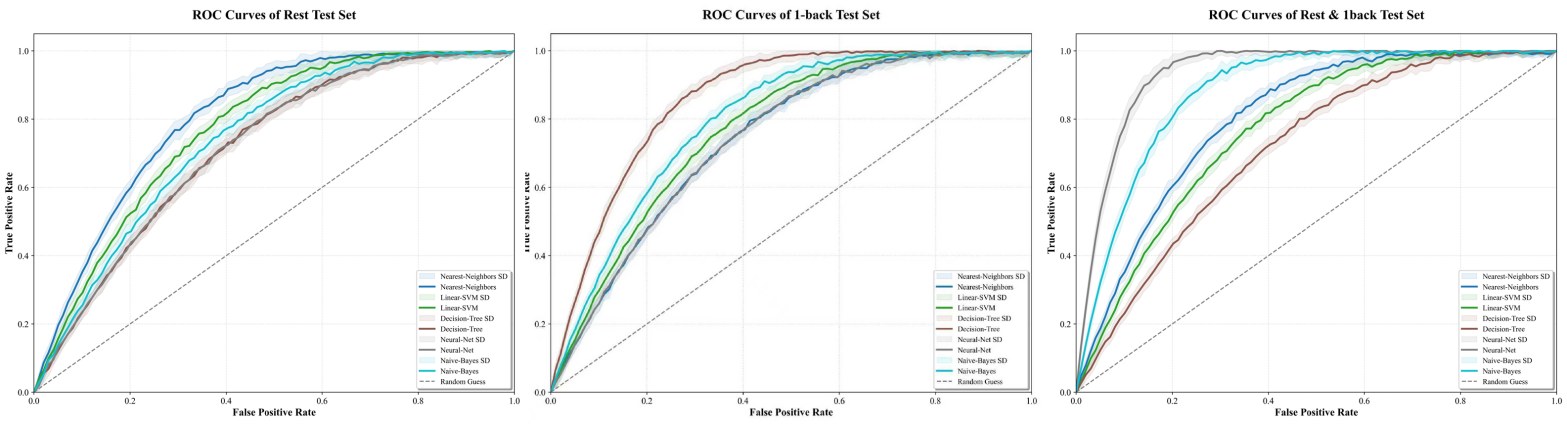
ROC curves of the proposed models on the rest, 1-back, rest and 1back test sets.

**Figure 9:**
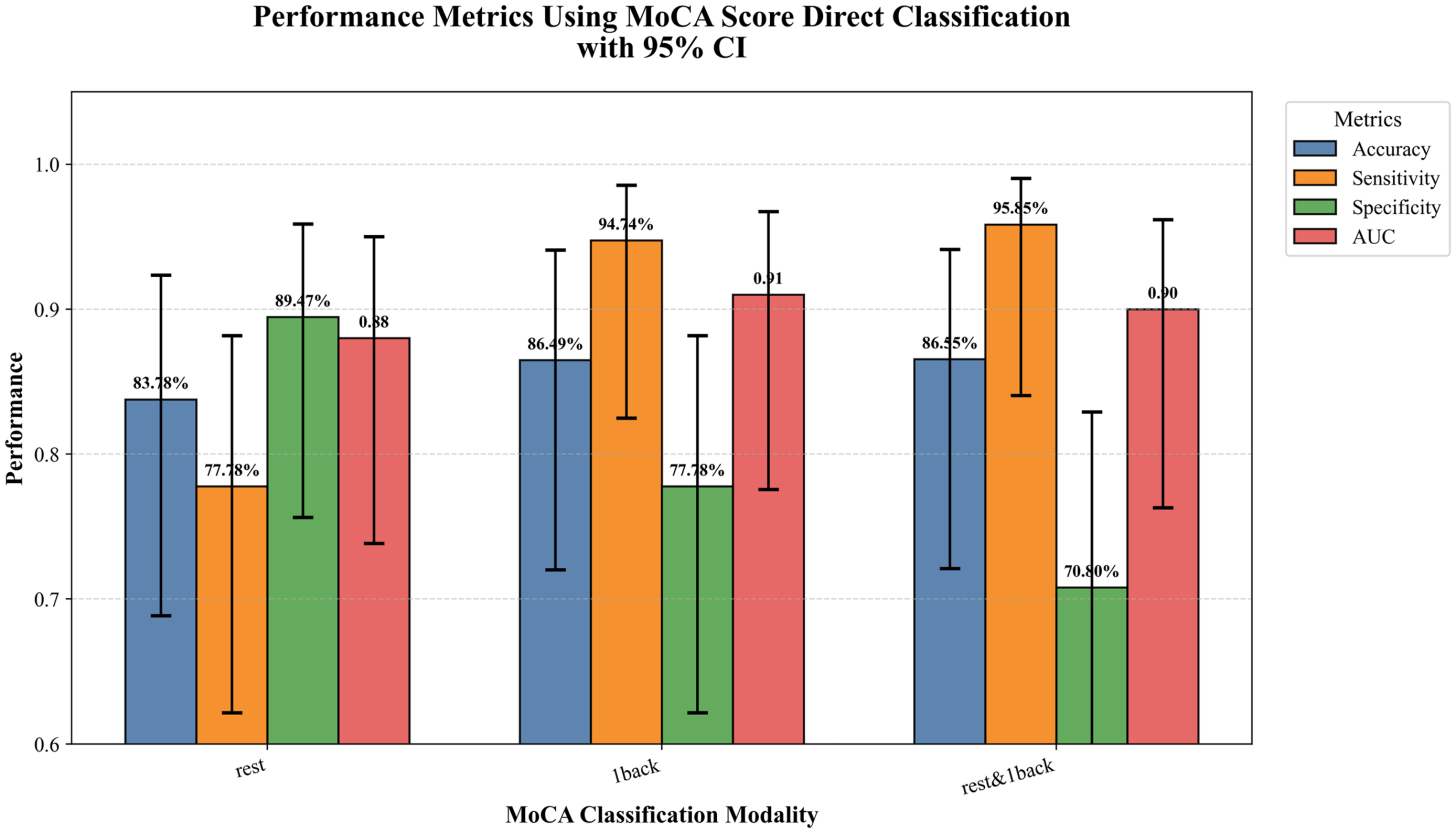
Comparison of neural network model and MoCA.

**Figure 10:**
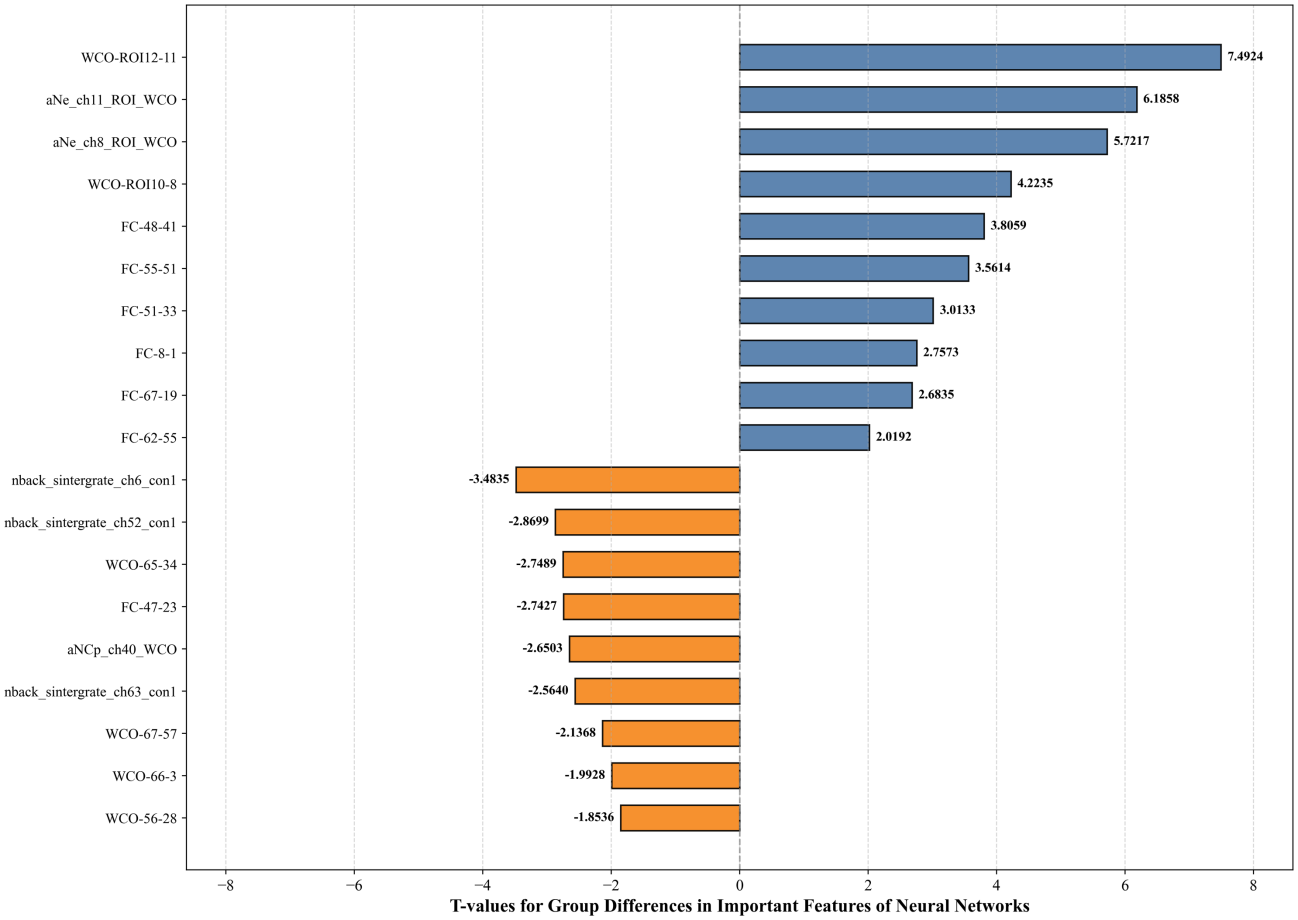
*T*-values for group differences in important features of neural networks.

Based on three datasets: resting-state, 1-back task, and an integrated dataset combining both feature sets, we evaluated the performance of five classifiers. For the resting-state dataset, the Nearest Neighbor model achieved an accuracy of 70.27%, with a sensitivity of 72.22% and specificity of 68.42% (see Figure 5). For the 1-back task dataset, the Decision Tree model showed an accuracy of 75.68%, sensitivity of 78.95%, and specificity of 72.22% (see Figure 6). The integrated dataset, combining resting-state and 1-back task features, showed the best results across all models. The Neural Network achieved an accuracy of 86.49%, sensitivity of 94.74%, and specificity of 77.78% (see Figure 7). ROC analysis further confirmed the superior discriminative ability of the Neural Network in the integrated dataset, with an AUC of 93.49%, as illustrated in Figure 8. Feature importance analysis revealed that the WCO between the right DLPFC and the right FPA as the most important feature, contributing the most to the model’s performance for early MCI detection. Other key indicators with considerable weights included WCO, FC, and Sintegrate, each playing a crucial role in capturing both brain connectivity and task-related activity. These features highlighted disruptions in brain regions like the right temporal lobe, DLPFC, and frontal pole, and their integration significantly improved the model’s diagnostic accuracy. Using MoCA scores as a threshold for group classification resulted in an accuracy of 86.55%, a sensitivity of 95.85%, and a specificity of 70.81% (see Figure 9), which was comparable to the performance of machine learning models based on individual paradigms (resting state or 1-back task). However, the integrated machine learning model, which combined features from both paradigms, demonstrated superior performance, highlighting the advantage of multimodal feature integration for early MCI detection. Table 2 summarizing the initial and optimized hyperparameters for the Neural Network. Additionally, we computed the group differences for these features (see Figure 10), and found that the most discriminative feature in the resting-state paradigm was the WCO between the right DLPFC and the right FPA, while for the 1-back task, it was the Sintegrate of channel 6, located in the left FPA. These findings further underscore their discriminative power and corroborate the feature contribution analysis.

**Table 2:** Initial and optimized hyperparameters of neural network model.

Hyper parameter	Initial values	Optimized value
batch_size	[8,16,32,64]	8
learning_rate	[0.001,0.005,0.01,0.05]	0.005
num_epochs	[10:5:100]	85
weight_decay	[0.001:0.01:0.1]	0.037
layer_1	[16,32,64]	[64]
layer_2	[4,8,16]	4

## 4 Discussion

The objective of this study is to develop an early screening model for Mild Cognitive Impairment (MCI) based on large-sample fNIRS prefrontal data, aiming to enhance the accuracy and efficiency of early MCI diagnosis. To achieve this goal, we collected characteristic indicators from 435 subjects during both resting state and n-back task state. Ultimately, we propose utilizing the combined characteristic indicators of resting state and n-back tasks to identify MCI patients, achieving an accuracy of 86.49%, a specificity of 77.78%, and a sensitivity of 94.74%. These metrics are significantly higher than those obtained using resting state or n-back task state alone. Moreover, the diagnostic model constructed by combining both states shows significant consistency when compared with the MoCA. This study represents the large-sample fNIRS data-driven neuroimaging research for MCI screening (39–41), this study also developed a machine learning model for MCI diagnosis by integrating features from both n-back and resting-state conditions, using a dedicated ensemble learning approach. The diagnostic model developed in this study has achieved both accuracy and specificity above 80%, demonstrating high diagnostic value and significantly surpassing previous research efforts (42–44).

fNIRS as a novel non-invasive brain imaging technology, has been widely applied in the early screening of Mild Cognitive Impairment (MCI) in recent years. Numerous studies have found that fNIRS holds great potential for the early diagnosis of MCI (45, 46). Among these, the n-back task state is commonly used for data collection. The n-back task is a classic paradigm for assessing working memory and cognitive load. For MCI patients, the decline in working memory capacity is one of the significant manifestations of impaired cognitive function. Therefore, the n-back task can effectively activate the prefrontal cortical brain regions associated with working memory (47, 48). However, our research found that the diagnostic model constructed solely based on the n-back task paradigm has an accuracy of 0.76 and a sensitivity of 0.79, indicating low diagnostic performance. Resting-state data can effectively reflect the overall and regional spontaneous neural activities of the brain. By analyzing functional connectivity (FC) and fractional amplitude of low-frequency fluctuations (fALFF), it reveals the intrinsic connectivity and functional changes in the brain networks of MCI patients (49). Combining n-back and resting-state fNIRS data can integrate brain functional information across different dimensions. This integration of multimodal data can provide richer information, improving diagnostic accuracy and reliability. In previous studies, we have not seen research that combines the n-back and resting-state task paradigms to construct a diagnostic model. Our research indeed confirms that the diagnostic performance is highest when both are combined.

To meet the demands of clinical diagnosis, it is crucial to transform neuroimaging data into a format that facilitates clinical decision-making. With advancements in machine learning algorithms and the continuous updating of data, artificial intelligence has been widely applied in disease diagnosis and the exploration of complex medical issues. In recent years, researchers have begun utilizing machine learning to identify AD or MCI. Dalin Yang employed resting-state fNIRS to construct an MCI diagnostic model using graph theory analysis and traditional machine learning methods with transfer learning. The results showed that the model accuracy was 81.27% for 30-s data and 76.73% for 90-s data. Keles et al. (42) collected resting-state prefrontal cortex fNIRS data from AD patients and constructed a diagnostic model using a support vector machine (SVM), achieving a sensitivity of 76% and a specificity of 68%. So-Hyeon used the 2-back task, Stroop task, and verbal fluency task to detect MCI and healthy individuals. They extracted metrics such as mean, integral, peak, slope, oxyhemoglobin, and deoxyhemoglobin from channels and regions of interest (ROIs), and classified the data using linear discriminant analysis (LDA) and SVM (42). The results revealed cognitive differences between the two groups in the ventrolateral prefrontal cortex. In terms of classification accuracy, the verbal fluency task achieved 80.77% (LDA) and 83.33% (SVM), the Stroop task achieved 79.49% (LDA) and 73.08% (SVM), and the 2-back task achieved 73.08% (LDA) and 69.23% (SVM). Zhang et al. (44) assessed functional connectivity in 71 channels and 9 ROIs by analyzing whole-brain resting-state fNIRS data from MCI patients and healthy individuals. The ROC curve (Receiver Operating Characteristic Curve) was calculated for functional connections with significant differences, and the AUC (Area Under the Curve) value was used as the evaluation standard for model classification. The results indicated decreased functional connectivity between the bilateral prefrontal, parietal, occipital, and right temporal lobes in the MCI group. The highest classification accuracy was observed for the connection between the right dorsolateral prefrontal cortex and the left occipital lobe, reaching 73.86%. These findings demonstrate the feasibility of using machine learning algorithms to construct models based on fNIRS feature values. However, the accuracy and specificity of diagnostic models in current studies remain relatively low, with poor stability. In contrast, this study integrates multimodal data, enriches the information collected, and significantly improves the accuracy and reliability of the diagnostic model. This cross-paradigm feature integration enables our model to capture a more comprehensive spectrum of MCI-related neurophysiological alterations, thereby endowing it with stronger discriminative power compared to single-paradigm models. The present study enrolled 462 participants, with a training cohort of 373 individuals, constituting one of the largest-scale fNIRS investigations in this field to date. Relative to many prior studies with smaller sample sizes, this substantial cohort significantly mitigates model overfitting due to random factors or individual variability, ensuring that the extracted feature patterns are more representative of the population and possess greater statistical power. This also provides ample data support for sophisticated ensemble machine learning algorithms. Regarding the machine learning methodology, this study adopted a systematic pipeline encompassing feature engineering and model optimization. First, through ensemble-based feature selection, the most discriminative subset of 108 features was robustly identified. Subsequently, during the classification stage, a neural network model demonstrated optimal performance. Compared to traditional machine learning methods or simpler deep learning frameworks employed in other studies, our strategy is likely more adept at uncovering deep, discriminative patterns from high-dimensional, multimodal data.

Additionally, this study compares the validation set, test set, and MoCA for MCI diagnosis to further verify the clinical effectiveness of the fNIRS diagnostic model. The MoCA is an important tool for clinical screening of MCI, but it is susceptible to influences such as age, education level, and errors from repeated measurements. Current research results indicate that the accuracy of the fNIRS model in MCI screening is comparable to that of the MoCA. Notably, the sensitivity and specificity of the MCI diagnostic model constructed by combining n-back and resting-state feature indicators are significantly higher than those of the MoCA. The MCI diagnostic model built using fNIRS has the potential to serve as an alternative to the MoCA. In clinical practice, a complete MoCA assessment typically requires approximately 10–15 min, whereas the combined resting-state and 1-back task protocol can be administered in about 10 min, representing a lower time investment. Furthermore, fNIRS provides objective neural activity data that are not influenced by language or educational background. From a cost–benefit perspective, although the initial investment in fNIRS equipment exceeds that of scale-based assessments, it offers distinct advantages, including high reproducibility, objective results, and independence from assessor subjectivity.

Based on large-scale resting-state and n-back task fNIRS data, this study constructed an ensemble machine learning model for the early identification of MCI. The results demonstrate that the dual-paradigm feature-based model achieved optimal diagnostic performance, with sensitivity and specificity superior to those of the commonly used clinical MoCA assessment. This suggests that integrating multidimensional brain functional information can effectively enhance the objectivity and accuracy of MCI detection. However, this study still has certain limitations. Firstly, since the thickness of the skull and hair in the prefrontal region can minimize scattering and attenuation effects, extensive prior research has established that the prefrontal cortex, particularly the DLPFC and the FPA, is closely associated with impairments in working memory, executive function, and other cognitive domains in patients with MCI (50), the fNIRS signals collected in this study are measured only from the prefrontal cortex. However, it is crucial to acknowledge the limitations inherent in this spatially focused approach. Mounting evidence indicates that the pathological alterations in AD are not confined to the prefrontal cortex. The reduced functional connectivity of the DMN has been widely established as a significant neuroimaging biomarker (13). Furthermore, functional network abnormalities in parietal and temporal lobes are also closely associated with disease progression (51). The primary acquisition of signals from the prefrontal cortex in this study may fail to capture characteristic abnormalities at the whole-brain network level, which, to some extent, constrains a comprehensive understanding of the neural mechanisms underlying MCI and may overlook potential, complementary diagnostic biomarkers with greater efficacy.

Secondly, this study is a single-center investigation, and the developed model may harbor biases specific to the local population and clinical setting. Furthermore, the absence of longitudinal follow-up in our MCI cohort precludes an assessment of disease progression, thereby leaving the predictive utility of fNIRS for conversion to dementia unvalidated. Additionally, although the independent test set (*n* = 47) employed in this study provided essential preliminary validation of model performance, its relatively modest size may not adequately capture the full spectrum of demographic and clinical heterogeneity present in the broader population.

While the integrated model achieved a high sensitivity of 94.74% and an overall accuracy of 86.49%, the specificity of 77.78% warrants attention in large-scale clinical deployment. In population-based screening, a specificity of 77.78% would result in a certain proportion of false positives, potentially increasing the burden of follow-up evaluations for both patients and healthcare systems. We propose the fNIRS-based model can serve as the first-line screening tool to identify high-risk individuals with high sensitivity. Positive cases can then be further evaluated using a combination of neuropsychological tests and clinical assessments to improve the overall specificity of the diagnostic pathway. Future iterations of the model will integrate additional biomarkers to reduce false positives.

Future research should optimize the channel layout of the functional near-infrared spectroscopy (fNIRS) system, extend the detection range to brain regions associated with MCI progression (e.g., the parietal lobe and temporal lobe), and integrate multimodal imaging technologies simultaneously. These adjustments will enable us to further explore the association between whole-brain functional network abnormalities and MCI, thereby enhancing the pathological interpretability of the proposed model. To validate the model’s practical value and generalizability, we will also conduct multi-center studies involving long-term follow-up of MCI patients to verify the model’s capability in predicting the progression of MCI to dementia. Ultimately, through these optimization and validation efforts, the approach proposed in this study is expected to be translated into a clinically useful tool, providing an efficient and objective solution for early MCI screening.

## Data Availability

The raw data supporting the conclusions of this article will be made available by the authors, without undue reservation.
