# Supplementary material for: fNIRS-based early identification of mild cognitive impairment: a large-scale multi-paradigm study with ensemble machine learning models
Source: Front Neurol. 2026 Mar 3;17:1738099. doi: 10.3389/fneur.2026.1738099 (PMC12992212; doi:10.3389/fneur.2026.1738099)
Supplement: Supplementary file 1 [file Data_Sheet_1.pdf]

Paradigm	Signal	Analysis Level	Feature Category	Feature Name	Description / Calculation	Tool / Software
Task-based	HbO	Channel / ROI	Activation (GLM)	Beta value	GLM-derived regression coefficient using canonical HRF convolution	NIRS-KIT toolbox
			Hemodynamic metric	Mean	Average HbO amplitude during 32s task block	Matlab scripts
				Integration	Area under the HbO curve within the 32s task time window	
				Peak amplitude	Maximum HbO value during 32s task period	
				Slope	Least-squares slope of the HbO signal from 2–7 s after task onset	
Resting-state	HbO	Channel / ROI	Functional connectivity	Pearson correlation	Time-domain correlation between HbO signals	NIRS-KIT
				Wavelet coherence (WCO)	Frequency-specific connectivity (0.01–0.08 Hz) based on wavelet-transformed signals	wcoherence(MATLAB built-in)
			Graph metric	Degree	Number of functional connections of a node	GRETNA toolbox
				Clustering coefficient	Local clustering of functional network	
				Global efficiency	Network-wide information integration efficiency	
				Local efficiency	Fault tolerance and local efficiency of the network	
				Degree centrality	Relative importance of a node in the network	
				Betweenness centrality	Fraction of shortest paths passing through a node	
				Shortest path length	Average shortest communication path between nodes	
				Small-world properties	Balance between segregation and integration in the network	

Table S1 Summary of Extracted fNIRS Features
